# Hybrid hydrogel system integrating thermoresponsive microspheres and KGN-loaded nanofibers enables efficient cartilage matrix regeneration

**DOI:** 10.1093/rb/rbag060

**Published:** 2026-03-21

**Authors:** Jian Huang, Chen Tang, Ye Wu, Tao Xiang, Keyi Chen, Xiaogang Bao, Bo Wang, Jie Ren, Jingbo Yin, Shifeng Yan, Guohua Xu

**Affiliations:** Department of Orthopedic Surgery, Spine Center, Changzheng Hospital, Naval Medical University, No. 415 Fengyang Road, Huangpu District, Shanghai 200003, China; Department of Polymer Materials, School of Materials Science and Engineering, Shanghai University, No. 99 Shangda Road, Baoshan District, Shanghai 200444, China; Department of Orthopedic Surgery, Spine Center, Changzheng Hospital, Naval Medical University, No. 415 Fengyang Road, Huangpu District, Shanghai 200003, China; Department of Orthopedic Surgery, Spine Center, Changzheng Hospital, Naval Medical University, No. 415 Fengyang Road, Huangpu District, Shanghai 200003, China; Department of Orthopedic Surgery, Spine Center, Changzheng Hospital, Naval Medical University, No. 415 Fengyang Road, Huangpu District, Shanghai 200003, China; Department of Orthopedic Surgery, Spine Center, Changzheng Hospital, Naval Medical University, No. 415 Fengyang Road, Huangpu District, Shanghai 200003, China; Department of Polymer Materials, School of Materials Science and Engineering, Shanghai University, No. 99 Shangda Road, Baoshan District, Shanghai 200444, China; Department of Polymer Materials, School of Materials Science and Engineering, Shanghai University, No. 99 Shangda Road, Baoshan District, Shanghai 200444, China; Department of Polymer Materials, School of Materials Science and Engineering, Shanghai University, No. 99 Shangda Road, Baoshan District, Shanghai 200444, China; Department of Polymer Materials, School of Materials Science and Engineering, Shanghai University, No. 99 Shangda Road, Baoshan District, Shanghai 200444, China; Department of Orthopedic Surgery, Spine Center, Changzheng Hospital, Naval Medical University, No. 415 Fengyang Road, Huangpu District, Shanghai 200003, China

**Keywords:** injectable hydrogel, thermoresponsive microspheres, KGN-loaded nanofibers, porosity engineering, cartilage regeneration

## Abstract

Articular cartilage has limited self-repair ability, and effective therapies for defects remain challenging. Injectable hydrogels are attractive for cartilage tissue engineering as they can fill irregular defects and mimic the native extracellular matrix (ECM). However, current hydrogels often suffer from dense microstructures, poor mechanical stability and inadequate biological signaling, restricting tissue regeneration. In this study, a multifunctional injectable hydrogel system based on oxidized dextran and carboxymethyl chitosan (ODex/CMCS) was developed by integrating degradable gelatin microspheres (GMs) and kartogenin-loaded cross-linked gelatin fibers (KGN@GFs). The GMs served as thermosensitive, dissolvable porogens that generated an interconnected porous structure within the hydrogels to enhance nutrient diffusion, cell infiltration and tissue ingrowth, while the KGN@GFs provided sustained KGN release and additional mechanical reinforcement. The resulting composite hydrogels exhibited favorable injectability, self-healing capability and a porous architecture conducive to cell survival and spatial organization. *In vitro* experiments demonstrated good cytocompatibility and significantly enhanced chondrogenic differentiation of bone marrow mesenchymal stem cells (BMSCs). Furthermore, implantation in rabbit cartilage defect models revealed that the ODex/CMCS/KGN@GFs/GMs hydrogel effectively promoted hyaline-like cartilage regeneration and integration with surrounding tissue. Collectively, this work presents a structurally and functionally optimized injectable hydrogel system that synergistically combines biochemical and structural cues to promote cartilage repair.

## Graphical Abstract

**Graphical Abstract:**
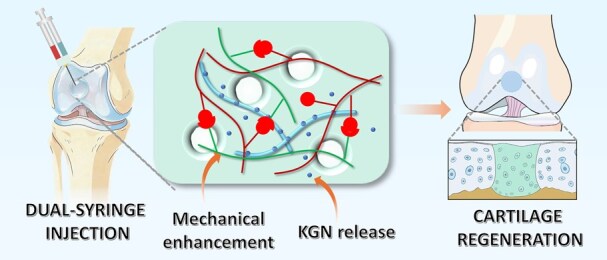

## Introduction

Articular cartilage (AC) plays a crucial role in weight-bearing, mechanical stress resistance and shock absorption. However, once damaged, cartilage’s ability to self-repair is limited due to factors such as the absence of blood vessels and restricted migration of chondrocytes [1]. Traditional cartilage repair methods often involve multiple surgeries, carry high risks of complications and face challenges in regenerating fully functional cartilage tissue [2]. In recent years, tissue engineering has introduced new approaches for cartilage defect repair [3]. Hydrogels, as artificial scaffold materials resembling the extracellular matrix (ECM), have garnered widespread attention in the field of tissue engineering. Currently, traditional hydrogels applied in clinics often function as passive, single-component fillers, but their efficacy is frequently limited by the requirement for invasive surgical implantation [4, 5]. Injectable hydrogels can overcome the limitations of traditional pre-formed scaffolds, enabling the minimally invasive filling of complex-shaped defects through microsurgery [6].

Scaffold materials in tissue engineering should feature a continuous porous structure and high porosity to provide sufficient space for cell growth, adhesion, proliferation and the secretion of extracellular matrix. Most traditional hydrogels possess nanoscale pores restricted by cross-linking density, which limits nutrient and oxygen transport and thus hinders cell proliferation, differentiation and ECM secretion [7]. In contrast, porous hydrogels facilitate nutrient and metabolite transport, promote cell proliferation and migration and more closely resemble the native ECM, thereby exhibiting enhanced biocompatibility [8]. Therefore, porous hydrogels are considered ideal materials for cartilage tissue engineering scaffolds.

Currently, various strategies have been developed to fabricate porous hydrogels, including phase separation [9], 3D printing [10] and particle leaching [11]. Among these approaches, particle leaching is particularly suitable for injectable hydrogels due to its simplicity and compatibility with *in situ* gelation. Salt particles are the most commonly used porogens; however, their application is limited by local cytotoxicity arising from high osmotic pressure during dissolution [12]. Other biodegradable microspheres often require prolonged degradation periods or specific enzymatic conditions for effective pore formation, which may compromise the controllability of pore generation *in vivo* [13, 14]. Gelatin, a denatured collagen derivative, exhibits excellent biocompatibility, low immunogenicity and predictable degradability under physiological conditions [15]. Gelatin microspheres (GMs) can be fabricated at low temperatures and rapidly dissolve at body temperature, enabling efficient *in situ* pore formation within injectable hydrogels without additional chemical or enzymatic triggers [16]. The resulting interconnected porous structures are expected to facilitate cell infiltration, nutrient diffusion, ECM secretion and subsequent tissue formation [17, 18].

However, the microstructure formed inside hydrogels often leads to a decrease in hydrogel mechanical strength and even structural collapse [19]. Although reducing the leaching agent’s dosage and increasing the hydrogel network’s cross-linking density can improve the mechanical strength of hydrogels, it may also lead to inadequate pore connectivity within the hydrogels [20]. Therefore, hydrogels need to be combined with other materials to enhance the hydrogels without affecting the diffusion of nutrients [21]. Fibers are commonly used materials to reinforce hydrogels. Zhuo *et al*. [22] developed a bamboo-inspired nanofiber assembly strategy to construct ultra-strong composite hydrogels, demonstrating that optimizing fiber–matrix interface interactions can drastically enhance mechanical performance. Similarly, Cai *et al*. [23] reported an injectable fiber-reinforced chitosan hydrogel system, in which the incorporation of interface-bonded nanofibers not only improved structural stability but also fostered a favorable microenvironment for cartilage regeneration. Cross-linked gelatin fibers (GFs) exhibit excellent biocompatibility and possess tunable biodegradability under physiological conditions. Gelatin fibers can be prepared by electrospinning, cross-linked and then introduced into the hydrogels to enhance their mechanical properties.

**Figure 1:**
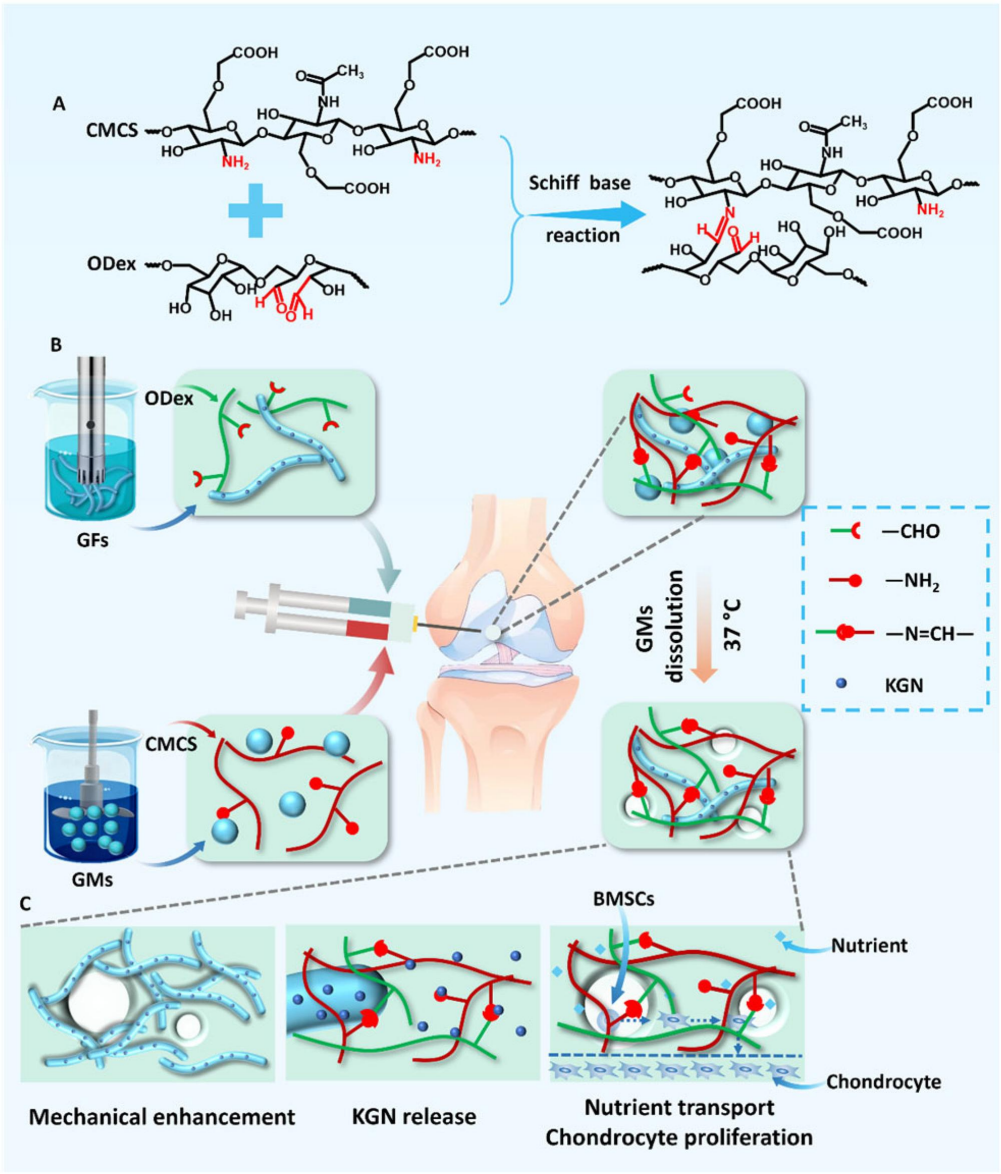
Schematic illustration of the preparation of nanofiber composite *in situ* pore-forming injectable hydrogel for cartilage tissue regeneration. (**A**) The hydrogel was formed through Schiff-base cross-linking between ODex and CMCS. (**B**) Mixing GFs/ODex and GMs/CMCS suspensions resulted in the formation of nanofiber/microsphere composite hydrogels, within which subsequent dissolution of the gelatin microspheres (GMs) created a porous structure. (**C**) GFs provided mechanical reinforcement and sustained KGN release, while the pores facilitated nutrient transport and chondrocyte proliferation. This synergistic design ultimately promotes cartilage repair.

While recent injectable fiber-reinforced hydrogels have demonstrated improved mechanical stability for cartilage repair [22], constructing a system that simultaneously achieves highly interconnected microporosity and sustained biochemical induction within a load-bearing matrix remains a critical challenge. In this study, to address this limitation, we developed a multifunctional hybrid hydrogel system based on oxidized dextran (ODex) and carboxymethyl chitosan (CMCS). We strategically employed temperature-responsive GMs as sacrificial templates to create a hierarchical porous architecture. This design overcomes the limited permeability of conventional bulk hydrogels [7] and is engineered to facilitate deep bone marrow mesenchymal stem cell (BMSC) infiltration and tissue integration [24]. Simultaneously, cross-linked gelatin nanofibers (GFs) loaded with kartogenin (KGN) were integrated to construct a biofunctional interface that ensures synchronized mechanical support and long-term biochemical induction (Figure 1) [25, 26]. The physicochemical properties of the composite hydrogels were systematically characterized. Crucially, biological evaluations demonstrated that this orchestrated system effectively promoted the chondrogenic differentiation of BMSCs *in vitro* and facilitated the regeneration of high-quality hyaline cartilage in a rabbit knee defect model.

## Materials and methods

### Materials

Carboxymethyl chitosan (CMCS) (*M*_w_ = 200 kDa) was purchased from Dalian Meilun Biotechnology Co., Ltd. 5-Fluorescein isothiocyanate (FITC) was obtained from Shanghai Macklin Biochemical Co., Ltd. Hexafluoro-2-propanol (HFIP), phthalic anhydride, 4-aminobiphenyl and 4-dimethylaminopyridine were purchased from Shanghai Aladdin Co., Ltd. Dextran (Dex, *M*_w_ = 40 kDa), sodium periodate, ethylene glycol, hydroxylamine hydrochloride, sodium hydroxide, hydrochloric acid, gelatin, ethyl acetate, anhydrous ethanol acetone, 1,4-dioxane, Na_2_HPO_4_·12H_2_O, NaH_2_PO_4_ and NaCl were all purchased from Sinopharm Group Chemical reagent Co., Ltd.

### Synthesis of oxidized dextran

ODex was synthesized via sodium periodate oxidation. Dextran (5 g, 0.0309 mol) was dissolved in 50 mL of deionized water, followed by the addition of 20 mL of an aqueous sodium periodate solution (3.3 g, 0.0154 mol). The reaction mixture was stirred in the dark at room temperature for 6 h. Subsequently, the excess oxidant was quenched by ethylene glycol (2.6 mL, 0.0463 mol) and stirring was continued for another 0.5 h. The resulting solution was transferred into a dialysis membrane (7000 Da) and dialyzed against deionized water for 3 days, with the water being replaced every 2–3 h. Finally, ODex was obtained after freeze-drying.

### Preparation of gelatin microspheres

GMs were prepared via the emulsion method. Briefly, 10-mL ethyl acetate and 30-mL gelatin solution (0.1 g/mL) were emulsified using a high-shear emulsifier at 19 000 rpm. The resulting emulsion was immediately poured into 50-mL soybean oil under stirring at 480 rpm. The mixture was maintained in an ice bath with continuous stirring for 15 minutes. Subsequently, the emulsion was transferred into 600 mL of pre-cooled ethanol (−20°C) and allowed to solidify for 10 minutes. The solidified microspheres were then washed three times each with 1,4-dioxane and acetone to remove residual soybean oil. Finally, the product was vacuum-dried at room temperature for 24 h, and GMs with a size range of 150–200 μm were collected by sieving through 60–300 mesh sieves.

### Synthesis of kartogenin

The synthesis of KGN was carried out according to the procedure described by Johnson *et al*. [27]. Phthalic anhydride (0.45 g), 4-aminobiphenyl (0.5 g) and 4-dimethylaminopyridine (0.0361 g) were dissolved in 100 mL chloroform/THF mixture (v/v = 3:1). The resulting mixture was stirred overnight at room temperature and then washed twice with brine. The aqueous phase was further extracted with ethyl acetate. The combined organic phases were dried over anhydrous sodium sulfate, concentrated under reduced pressure and purified by flash chromatography. Successful KGN synthesis was confirmed by ^1^H NMR analysis (Supplementary Figure S1), with characteristic peaks being observed for the benzene ring (*δ* = 7.34, 7.46 ppm), the methine proton adjacent to the carboxyl (*δ* = 7.90 ppm) and the imino proton (*δ* = 10.50 ppm).

### Preparation of cross-linked GFs

Gelatin was dissolved in HFIP to prepare a spinning solution at a concentration of 0.1 g/mL. The solution was loaded into a 5-mL syringe, which was then mounted on a syringe pump equipped with a 17-gauge needle. The electrospinning process was carried out under the following parameters: positive voltage of +6 kV, negative voltage of −2 kV, a collection distance of 6 cm and a flow rate of 10 mL/h. The resulting GF membranes were dried under vacuum at room temperature for 24 h. KGN solution (0.2 g/mL in dimethyl sulfoxide, DMSO) was mixed with the gelatin spinning solution at a volume ratio of 1:20 to prepare KGN-loaded fiber membranes, and electrospinning was performed under the same conditions.

The GF membranes were then immersed in ethanol, followed by fragmentation into short fibers using a high-shear emulsifier at 19 000 rpm for 20 minutes and cross-linked with glutaraldehyde. Then, the ethanol was removed via high-speed centrifugation at 12 000 rpm. The resulting cross-linked gelatin short fibers (GFs) were vacuum-dried and stored for subsequent use.

### Preparation of fluorescent GMs

FITC-labeled gelatin was prepared through a conjugation reaction between the isothiocyanate group of FITC and the amino groups of gelatin. Gelatin solution (100 mL, 0.05 g/mL) and FITC solution (50 mL, 0.01 mg/mL) were mixed, and the pH was adjusted to 8.5 using 0.1 M NaOH, then reacted at 40°C for 8 h in the dark. Subsequently, the reaction mixture was dialyzed against deionized water using a dialysis membrane (7000 Da) for 3 days, with the water being changed every 2–3 h. Finally, FITC-labeled gelatin was obtained by freeze-drying [1]. FITC-labeled GMs were subsequently fabricated using the emulsion method described above.

### Preparation of nanofiber composite *in situ* pore-forming hydrogels

GFs (6 wt%) and GMs (6 wt%) were separately dispersed in ODex and CMCS solutions, respectively. The resulting GFs/ODex and GMs/CMCS suspensions were thoroughly mixed. A nanofiber-reinforced *in situ* pore-forming hydrogel was then formed using a dual-syringe injection system. The final composite hydrogel contained 3 wt% GFs, 3 wt% GMs and a total of 3 wt% of the ODex/CMCS mixture (–CHO:–NH_2_ = 1:1).

### Physicochemical characterization

The amino content of CMCS and the aldehyde content of ODex was measured using the potentiometric titration method. ODex and Dex were analyzed using a Fourier-transform infrared spectrometer in the region of 500–4000 cm^−1^. The microstructures of GMs, GFs and hydrogels were observed using a scanning electron microscope (SEM, SU1500, Japan). Changes in the size of GMs before and after swelling were examined using an optical microscope. The dissolution process of FITC-GMs in the hydrogels was monitored through fluorescence microscopy. In addition, the microstructure of GFs was observed using transmission electron microscopy (TEM, JEM2100F, Japan).

The dissolution of FITC-labeled gelatin was determined by measuring the absorbance at 495 nm using a UV-visible spectrophotometer. The concentration was then determined based on the FITC standard curve. The GMs content in the hydrogel was calculated using the following formula:


$$\mathrm{GMs}\;\mathrm{content}\;(\%)=\frac{\mathrm{FITC}\;\mathrm{content}\;\mathrm{in}\;\mathrm{hydrogel}\;-\;\mathrm{FITC}\;\mathrm{content}\;\mathrm{in}\;\mathrm{buffer}}{\mathrm{FITC}\;\mathrm{content}\;\mathrm{in}\;\mathrm{hydrogel}}\times 100\%$$

Rheological evaluation of cylindrical hydrogel samples (φ 12 mm × 2 mm) was conducted on a rotational rheometer maintained at 37°C. Measurements were performed at 0.1% oscillatory strain with a frequency sweep ranging from 0.1 to 100 rad/s.

Compression tests of cylindrical hydrogel samples (φ 10 × 10 mm) were performed on a 500 N universal testing machine at a speed of 5 mm/min.

### Porosity, swelling and degradation of hydrogels

First, the hydrogel was quantified in PBS for 3 days and lyophilized to obtain its dry weight (W_1_) and volume (V). Next, it was immersed in ethanol and subjected to vacuum soaking for 2 h. Finally, the wet weight (W_2_) was recorded after reaching saturation. The porosity was calculated using the following formula:


$$\mathrm{Porosity}=\frac{（W_{2}-W_{1}）/\rho }{V}\times 100\%$$

The density of ethanol is taken as 0.789 kg/m³.

The swelling ratio was determined by measuring the weight change of the hydrogel after equilibrium swelling in 0.03 M PBS at 37°C. The weights of the hydrogel at equilibrium swelling and in the freeze-dried state were recorded as $W_{S}$ and W_0_, respectively. The swelling ratio was calculated as follows:


SR=WSW0 ×100%

The degradation profile of the hydrogel was assessed by monitoring its mass loss over time. A hydrogel sample (400 μL) with an initial mass (*W*_0_) was incubated in 10 mL of 0.03 M PBS containing lysozyme (1 mg/mL) at 37°C in a constant-temperature shaker operating at 40 rad/min. The buffer solution was replaced weekly. At predetermined time intervals, the hydrogel was taken out, surface moisture was blotted away and its wet mass was recorded as $W_{t}$. The mass retention rate was calculated using the following formula:


$$\mathrm{WR}=\frac{W_{t}}{W_{0}}\times 100\%$$

### *In vitro* release behavior of KGN from hydrogels

The absorption spectrum of KGN in PBS was measured using a UV-Vis Spectrophotometer (Agilent 8453, USA), and a standard curve was established using a series of gradient concentrations. To evaluate the drug release profile, 5 mg of hydrogel was loaded into an ultrafiltration tube containing 4 mL of PBS with 0.5% Tween 80 at 37°C with shaking (60 rpm). The release medium was then collected at predetermined time points and quantified by UV spectrophotometry.

### Biocompatibility

Immortalized mouse BMSCs were commercially obtained for *in vitro* material compatibility experiments. Cell viability and cytotoxicity were evaluated using CCK-8 (Cell Counting Kit-8) assay and Live/Dead fluorescent staining.

To assess the effect of hydrogels on cells *in vitro*, a series of hydrogels (5 mm in length, 8 mm in diameter) with varying compositions was prepared. After irradiation and sterilization, the hydrogels were immersed in 2 mL of fresh cell culture medium containing 5% CO_2_ at 37°C. After 48 h, extracts from the immersed hydrogels were collected. Cell proliferation was assessed using CCK-8, while Live/Dead staining was performed to evaluate cellular activity.

Immortalized mouse BMSCs were inoculated in 100 μL of medium at 1 × 10³ cells/well and incubated overnight in a sterile 5% CO_2_ environment until the cells were adherent to the wall. All medium in the well plates was then replaced with the pre-prepared immersion extract medium. After incubation for 12 h in a standard cell culture environment, 10 μL of CCK-8 solution was added, followed by another 2 h of incubation. The absorbance was measured at 450 nm using a microplate reader. The same procedure was performed at 24, 48 and 72 h, respectively.

Cell viability was further evaluated using the Viability/Cytotoxicity Assay Kit with calcineurin-AM (green) and Propidium (PI) (red) staining (Invitrogen, L3224, USA). To observe cell growth in hydrogels, cells were inoculated on hydrogels in 24-well plates and cultured for 3 days. Cells cultured within hydrogels stained with calcein AM and ethidium bromide (TCS SP8; Leica, Wetzlar, Germany) were photographed by confocal laser scanning microscopy and evaluated in stereomicroscope images. The hydrogels loaded with cells were then dehydrated and dried using an ethanol gradient (50–60–70–80–90–95–100%) before observation by SEM.

### Chondrogenic differentiation *in vitro*

Mouse BMSC were obtained from femurs of 8-week-old male C57/BL 6 mice for *in vitro* chondrogenic differentiation experiments. Briefly, bone marrow was gently flushed out from the femoral cavity with a 1-mL hypodermic syringe and cultured in complete medium [Dulbecco’s Modified Eagle’s Medium (DMEM)/F12 with 10% fetal bovine serum and 1% penicillin-streptomycin]. The growth medium was changed every 2 days, and third-generation BMSCs were used for experiments.

Plates were spread at a density of 1.2 × 10^6^ mL^−1^, and the composite hydrogels (10 mm in length and 8 mm in diameter) were co-cultured with BMSCs in chondrogenic differentiation-inducing medium after the cells were fully grown. The chondrogenic differentiation induction medium consisted of high glucose DMEM (Sigma-Aldrich) containing penicillin (100 U mL^−1^), streptomycin (100 μg mL^−1^), L-glutamine (2 mM), N-(2-hydroxyethyl)piperazine-29-(2ethane-sulfonic acid) (10 mM, Sigma-Aldrich) (all others from Life Technologies), insulin- transferrin-sodium selenite media supplement (ITS)+1 premix (Corning, Discovery Labware, Inc., MA, USA), 10 ng mL^−1^ transforming growth factor β3 (ProSpec, NJ, USA), 100 nM dexamethasone, 365 μg mL^−1^ ascorbic acid 2-phosphate and L-proline (40 μg mL^−1^) (all from Sigma-Aldrich). The medium was changed every 2 days.

After 3 weeks, cells were gently washed with PBS buffer, fixed with 4% paraformaldehyde and stained with Alcian Blue pathology stain. After the cells were co-cultured with hydrogel samples for 10 days, RNA was extracted using an RNA extraction kit and its concentration was determined using an ND-1000 spectrophotometer (Nanodrop Technologies). Then, an aliquot of RNA (100 ng) was reverse transcribed into complementary DNA. Real-time polymerase chain reaction (qPCR) was performed on an Applied Biosystems 7900 HT Fast real-time-PCR instrument. Relative gene expression was calculated using the ΔΔCT method, and fold differences were calculated using expression 2^−ΔΔCt^.

### Cartilage regeneration in the rabbit knee cartilage defect model

A cartilage defect model of the knee joint was established in 20 New Zealand rabbits of 2 months of age, which were randomly divided into five groups (*n* = 4 per group). After effective anesthesia, the rabbits were shaved and sterilized. A medial patellar incision was made to expose the knee joint, which was then dislocated laterally upon flexion. An osteochondral defect (Φ4 mm diameter, 1.5-mm depth) was created in the center of the trochlear groove using a cylindrical drill. The damaged osteochondral tissue was removed and subsequently filled with hydrogel. At 10 weeks post-surgery, the femoral condyles were harvested, fixed in 10% formalin and decalcified in ethylenediaminetetraacetic acid buffer. Tissue sections were then stained with hematoxylin and eosin (H&E) and Safranin O–Fast Green. Type II collagen (Col-II) expression in the defect area was assessed via immunohistochemical staining. Cartilage regeneration was quantitatively evaluated using a standardized scoring system (Supplementary Tables S1 and S2). All animal experiments were performed in accordance with institutional ethical guidelines and approved by the Animal Care and Use Committee of Naval Medical University (82272533).

### Statistical analysis

Data were expressed as mean ± standard deviation and analyzed statistically using SPSS 25.0 software. Unpaired Student *t*-test was used for comparison between the two groups. Analysis of variance using Bonferroni *post hoc* test. Significance was defined as a *P* value ≤ 0.05. All representative images of tissues or cells were selected from at least three independent experiments with similar results.

## Results and discussion

### Preparation of gelatin microspheres and gelatin fibers

**Figure 2:**
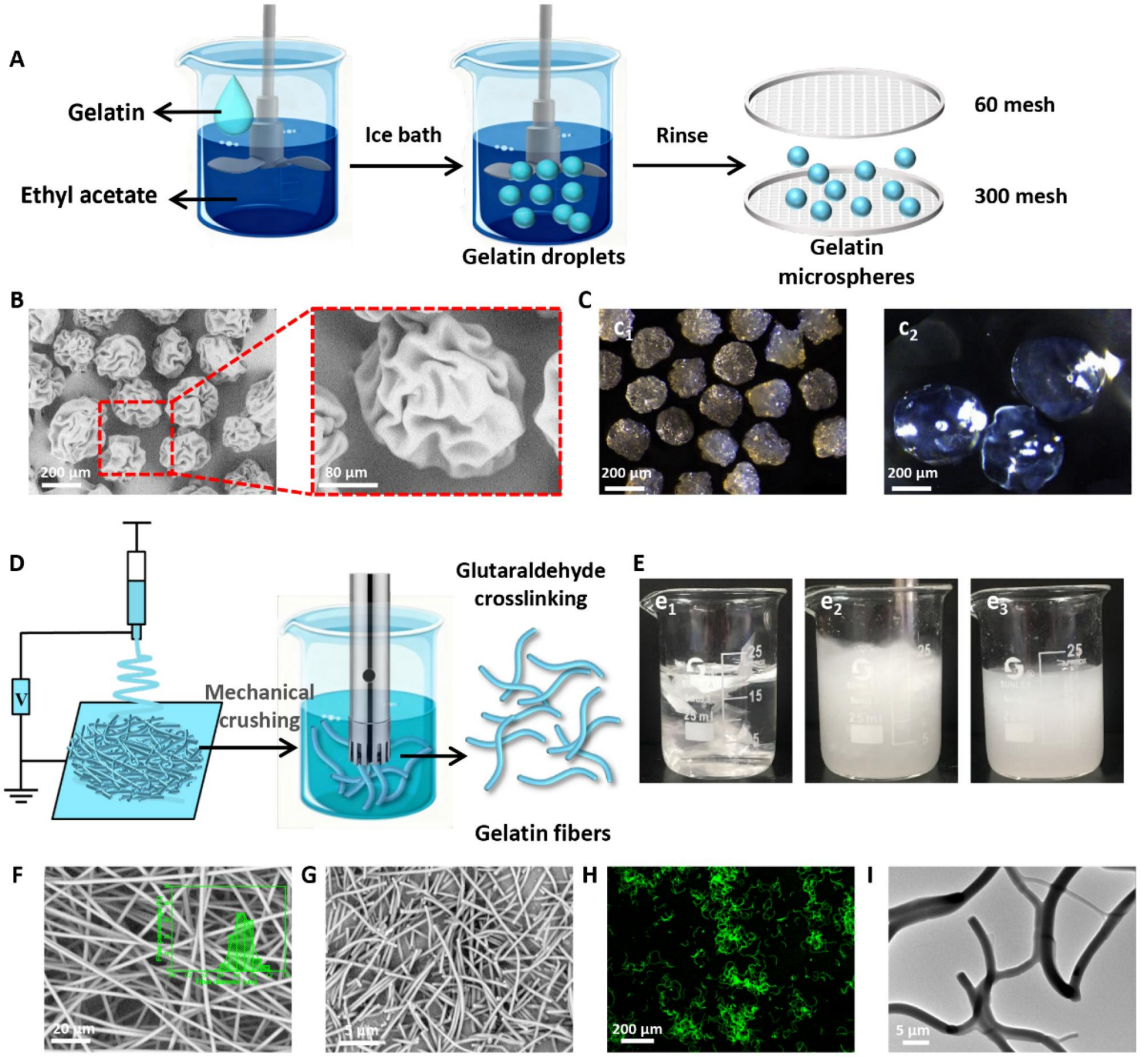
Preparation of GMs and GFs. (**A**) Schematic for the preparation of GMs. (**B**) SEM images of GMs. (**C**) Optical microscope images of GMs (**c_1_**) before and (**c_2_**) after swelling. (**D**) Schematic for the preparation of GFs. (**E**) Photograph of the preparation of GFs: (**e_1_**) before shearing, (**e_2_**) during shearing and (**e_3_**) after shearing. SEM images of GFs (**F**) before and (**G**) after crushing. (**H**) Confocal laser scanning microscopy and (**I**) transmission electron microscopy images of GFs after cross-linking.

GMs were prepared using a gelatin/ethyl acetate emulsion method (Figure 2A). After emulsification, gelatin droplets were solidified in an ice bath, followed by thorough washing. The resulting GMs were vacuum-dried at room temperature and sieved to achieve a size range of 150–200 μm. The GMs exhibited near-spherical morphology with uniform size distribution and surface wrinkling (Figure 2B). This wrinkling likely resulted from rapid moisture loss during the removal of soybean oil using 1,4-dioxane and acetone. The emulsion-derived GMs retained gelatin’s solubility at 37°C but only swelling without dissolving at room temperature. Stereomicroscopy confirmed that GMs maintained intact spherical structures after swelling in PBS (Figure 2C). Size analysis of GMs (Nano Measurer software) revealed an increase in average diameter from 184.9 μm (pre-swelling) to 378.0 μm (post-swelling) (Supplementary Figure S2). Previous studies indicate that scaffolds with pore sizes of 200–500 μm enhance chondrocyte viability and ECM production 30 [28, 29].

Gelatin fiber membranes were prepared by electrospinning, and uniformly dispersed short GFs were obtained after high-speed shearing (Figure 2D and E). An opaque GF dispersion was obtained after the GF membranes were processed with a high-shear emulsifier. These GFs were cross-linked with glutaraldehyde. SEM imaging exhibited GF membranes with a narrow diameter distribution (average: 870 nm) (Figure 2F). Fiber diameter distribution analysis showed uniform length distribution (9–30 μm, average: 17.34 μm) of GFs (Figure 2G). Confocal laser scanning microscopy and transmission electron microscopy revealed that the cross-linked GFs interlocked into a cross-stacked network (Figure 2H and I). When incorporated into hydrogels, this interconnected GFs network was expected to provide structural support and reinforcement. This dynamic pore evolution not only altered the physical architecture but also provided essential spatial freedom for BMSC migration and aggregation, addressing the confinement issues of traditional dense hydrogels.

### Construction of *in situ* pore-forming nanofiber composite injectable hydrogels

ODex was prepared using sodium periodate as the oxidant [30]. FTIR and ^1^H NMR confirmed the conversion of Dex into an open-chain form, ODex, which contained aldehyde groups in its macromolecular chain (Supplementary Figure S3). Potentiometric titration analysis determined that the aldehyde group content of ODex was 4.08 mmol/g, and the amino group content of CMCS was 3.38 mmol/g (Supplementary Figure S4).

**Figure 3:**
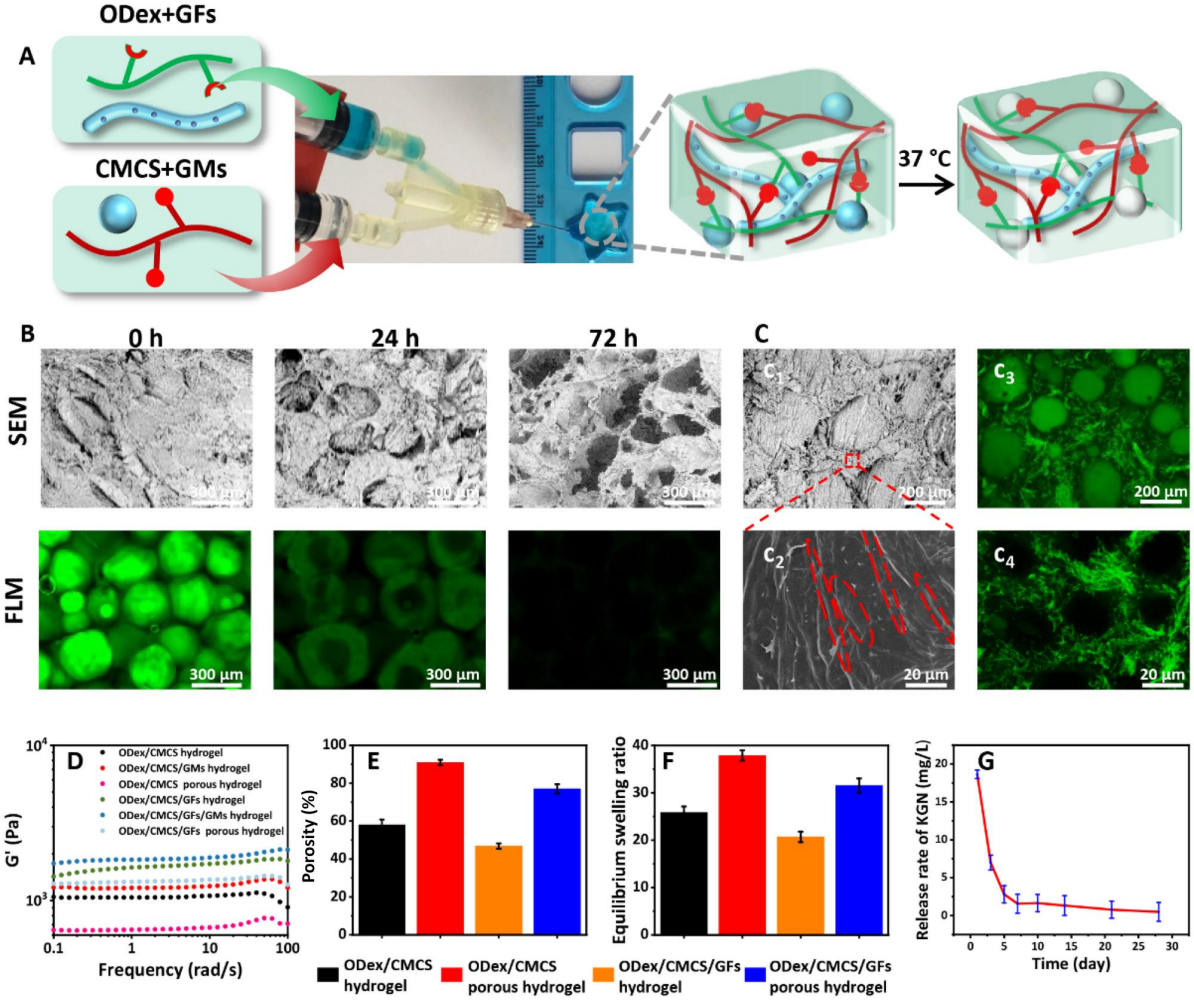
Preparation of nanofiber composite *in situ* pore-forming injectable hydrogels. (**A**) Schematic illustration for the preparation of *in situ* pore-forming injectable hydrogels. (**B**) SEM and fluorescence microscopy (FLM) of *in situ* pore-forming injectable hydrogels. (**C**) Morphology of the ODex/CMCS/GFs/GMs hydrogel (before pore formation) and the ODex/CMCS/GFs porous hydrogel (after pore formation). (**D**) Rheological properties of hydrogels. (**E**) Porosity of hydrogels. (**F**) Equilibrium swelling ratios of hydrogels. (**G**) KGN release kinetics from nanofiber composite *in situ* pore-forming hydrogels.

The fabrication of the *in situ* pore-forming nanofiber composited injectable hydrogels is illustrated in Figure 3A. The precursor solution can be injected into a mold to fabricate hydrogels with specific shapes. Mixing GFs/ODex and GMs/CMCS suspensions resulted in the formation of nanofiber/microsphere composite hydrogels. The gelation mechanism was attributed to the Schiff-base reaction between the amino groups in CMCS and the aldehyde groups in ODex [31]. Otherwise, GMs and GFs significantly accelerated gelation, reducing the time from 78.0 ± 15.1 to 26.9 ± 6.7 s (Supplementary Figure S5). As shown in Supplementary Figure S6, GMs dissolved completely within 3 days. Their dissolution produced an interconnected porous network in the hydrogel, resulting in the final nanofiber composite *in situ* pore-forming injectable scaffold. The pore-formation process was monitored by SEM and fluorescence microscopy (Figure 3B). No obvious dissolution was observed before 12 h. Partial dissolution became visible at 24 h, and complete dissolution occurred by 72 h, yielding a highly porous hydrogel structure. Fluorescence images of FITC-labeled GMs further confirmed the gradual dissolution from the interior of the microspheres, consistent with the SEM observations.

As shown in Figure 3C1 and C2, SEM confirmed a persistent cross-stacked fibrous architecture in the hydrogels after the dissolution of GMs. Fluorescence microscopy further revealed that the dissolution of GMs generated internal micropores, with GFs still dispersed throughout the interstitial spaces (Figure 3C3 and C4). This architecture not only enhanced the mechanical properties of hydrogels but also effectively prevented structural collapse. The effects of GMs and GFs on the mechanical properties were investigated using a rotational rheometer. Compared with ODex/CMCS hydrogel, the storage modulus (G′) of ODex/CMCS/GMs hydrogel increased from 1.0 to 1.3 kPa, while that of ODex/CMCS/GFs hydrogel rose to 1.5 kPa and ODex/CMCS/GFs/GMs hydrogel reached 1.7 kPa. Following dissolution of GMs (forming a porous structure), which generated a porous structure within the hydrogel, the hydrogel structure became looser, resulting in reduced G′ values of 0.6 kPa, and 1.3 kPa for ODex/CMCS/GMs and ODex/CMCS/GFs/GMs hydrogel systems, respectively (Figure 3D). The compression test results are consistent with the rheological results. Compared with the ODex/CMCS hydrogel, the compressive stress and strain of the ODex/CMCS/GMs hydrogel changed slightly from 94% and 1.12 MPa to 93% and 1.14 MPa, respectively. The ODex/CMCS/GFs hydrogel exhibited values of 93% and 1.12 MPa, while the ODex/CMCS/GFs/GMs composite hydrogel reached 92% and 1.17 MPa. Following the dissolution of GMs, the compressive stress and strain decreased to 91% and 1.04 MPa for ODex/CMCS porous hydrogel and to 91% and 1.13 MPa for ODex/CMCS/GFs porous hydrogels, respectively (Supplementary Figure S7). The superior mechanical strength exhibited by our nanofiber-incorporated *in situ* pore-forming hydrogels is consistent with the findings of Wang *et al*. [32], who demonstrated that the integration of a physically entangled nanomaterial network effectively dissipates energy and provides essential structural stability to porous architectures.

**Figure 4:**
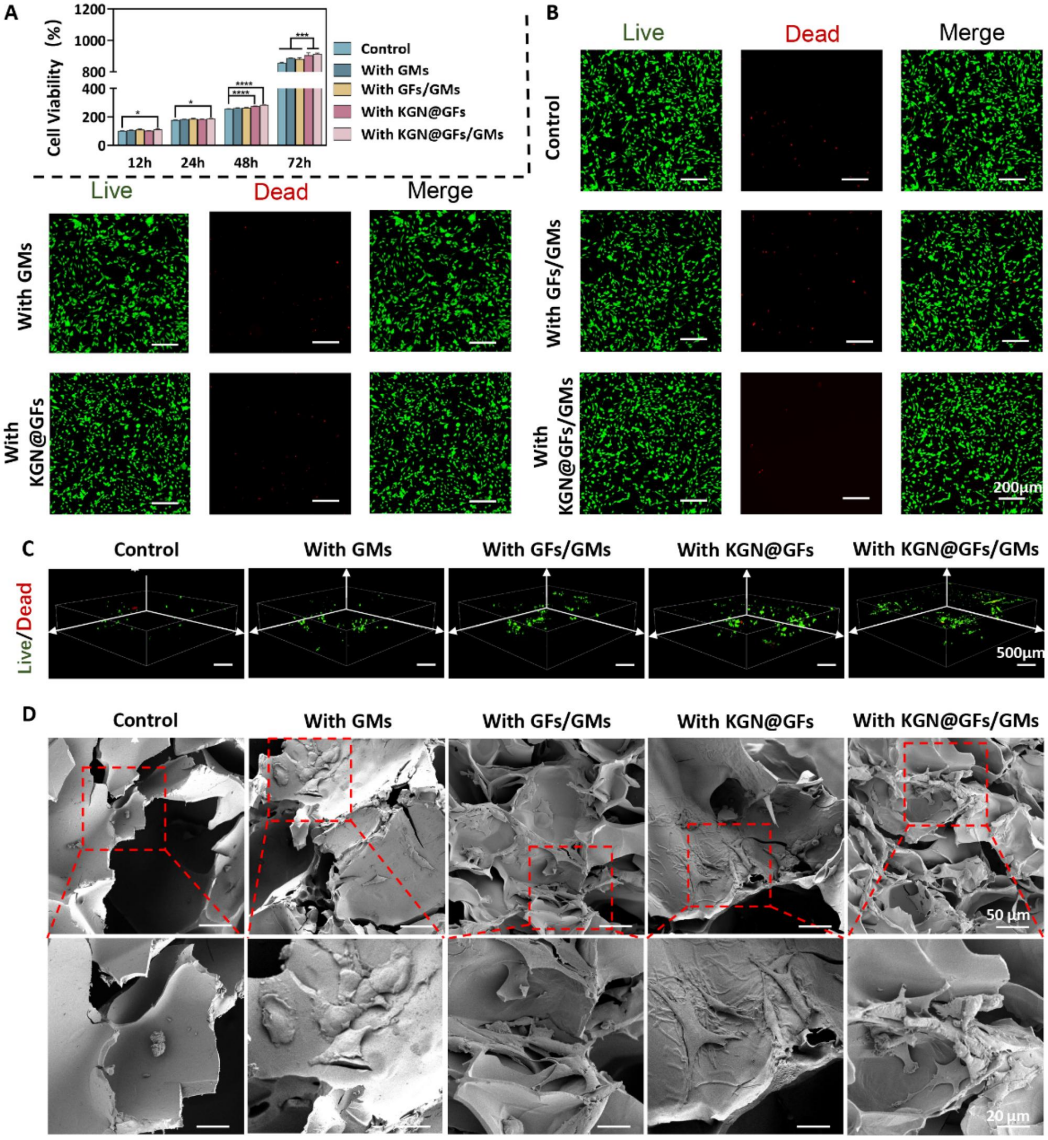
Biocompatibility of various hydrogels. (**A**)The cell viability of various hydrogels analyzed by CCK8 (mean ± SD; *P < 0.05, ***P < 0.001, ****P < 0.0001). (**B**) Images of BMSCs after 5 days of co-culture stained with Live/Dead. (**C**) Three-dimensional Live/Dead staining images of BMSCs seeded on the hydrogels. (**D**) Scanning electron microscopy (SEM) images of BMSCs cultured on the hydrogels.

The effects of GMs and GFs on the porosity and swelling behavior of the hydrogels were also investigated. Compared to the porosity of 58.0% for the ODex/CMCS hydrogel, the ODex/CMCS/GMs hydrogel exhibited a significant increase in the porosity to 91.0%. The ODex/CMCS/GFs hydrogel showed a reduced porosity of 46.8%, while the ODex/CMCS/GFs/GMs hydrogel demonstrated a porosity of 77.0% (Figure 3E). Compared to the ODex/CMCS hydrogel, after GMs dissolution, the ODex/CMCS/GMs hydrogel formed a looser 3D network structure, which increased its equilibrium swelling ratio from 25.9 to 37.9 (Figure 3F). Incorporating GFs into the ODex/CMCS hydrogel increased its solid content, resulting in a denser three-dimensional network and consequently reducing the swelling ratio to ∼18.5. Hydrogels containing both GMs and GFs maintained a moderate swelling ratio of ∼27.5. Similarly, the porous structure endowed the hydrogel with remarkable degradability, yielding a degradation rate of 81.5 ± 4.3% within 7 weeks (Supplementary Figure S8). Thus, porous structures formed upon GMs dissolution enhanced both the swelling ratio and porosity of scaffolds, whereas incorporating nanofibers into the hydrogel decreased these parameters [18, 33, 34].

KGN is a therapeutic agent that promotes the repair of osteochondral defects *in vivo*. The controlled release of KGN effectively enhances chondrogenic differentiation and the secretion of cartilage ECM, thereby facilitating the repair of cartilage defects *in vivo*. Figure 3G illustrates the release profile of the *in situ* pore-forming nanofiber composite hydrogels. An initial burst release of 18 mg/L was observed from the KGN-loaded hydrogel within the first day. Subsequently, the release reached 0.4 mg/L by day 28, which falls within the established effective concentration range (0.3–30 mg/L) as reported [35]. This concentration was expected to promote chondrocyte proliferation and upregulate chondrogenesis-related genes. Consistent with prior reports [36], nanofibers function as effective carriers for the sustained release of KGN. This sustained release profile maintains the bioactivity of KGN throughout the cartilage repair process.

In summary, the physicochemical characterization suggests that this hybrid system is not a static mixture but a dynamic scaffold. The synchronized process of GM dissolution and GF reinforcement addresses the traditional trade-off between high porosity and mechanical stability. This “mechanical skeleton” provided by the GFs ensures that the scaffold remains structurally intact during the *in situ* pore-forming process, establishing a stable physical foundation for the subsequent spatial orchestration of cell infiltration and tissue ingrowth.

### Loading and viability of BMSCs in hydrogels

We designed a series of hydrogels with different compositions for subsequent biological experiments (Supplementary Table S3). CCK-8 assays showed that all five hydrogel formulations supported BMSC metabolic activity from 12 to 72 h, with the KGN@GFs/GMs group producing the highest signal at 72 h (*P* < 0.001 versus ODex/CMCS) (Figure 4A). Extract-based Live/Dead staining confirmed negligible cytotoxicity for all formulations after 5 days (Figure 4B). 3D confocal Live/Dead imaging of cells seeded on the materials revealed deeper cell penetration and more homogeneous viable cell distributions in GM-containing hydrogels, particularly in the KGN@GFs/GMs hydrogel group, where cells frequently formed localized multicellular clusters within porous regions (Figure 4C). SEM micrographs corroborated these observations: unmodified ODex/CMCS hydrogel group presented sparse, rounded cells on a relatively smooth surface; the GMs-containing hydrogels displayed porous niches, supporting robust adhesion and local aggregation; while the GFs-containing hydrogels featured a relatively rough surface with enhanced support, facilitating improved cell spreading and adhesion (Figure 4D). To further evaluate the bioactive potential of the hydrogels in recruiting endogenous stem cells, a scratch assay was performed to monitor the migration of BMSCs. As shown in Supplementary Figure S9, the wound closure rate was significantly accelerated in the presence of functionalized hydrogels. Specifically, the ODex/CMCS/KGN@GFs/GMs group exhibited the fastest migration, with the wound area almost completely closed within 24 h.

These data indicate that all formulations are cytocompatible, and the incorporation of GMs substantially improved cell loading, penetration depth, local cell density and early metabolic activity. GMs acted as porogens, generating interconnected microporosity upon partial dissolution, thereby producing protected niches that favor cell colonization and multicellular aggregation—an effect previously documented for GMs and microsphere-templated hydrogels [37]. Moreover, the porous microarchitecture produced by GMs enhanced local mass transport (nutrient and waste exchange) [38, 39] compared with dense Schiff-base networks, thereby supporting higher local cell densities and sustained metabolic activity—consistent with the higher CCK-8 signal and more viable cells observed in GM-containing groups.

Although the effects of KGN on differentiation are discussed in detail elsewhere, it is generally biocompatible and has been reported to promote cell proliferation in biomaterial delivery systems [27, 40]; in our experiments, the KGN@GFs/GMs formulation yielded the strongest early metabolic activity and most pronounced multicellular clustering. These results suggest a synergistic effect of sustained KGN availability and the porous microenvironment generated by GMs, enhancing BMSCs’ survival and aggregation.

### *In vitro* chondrogenic differentiation capability

**Figure 5:**
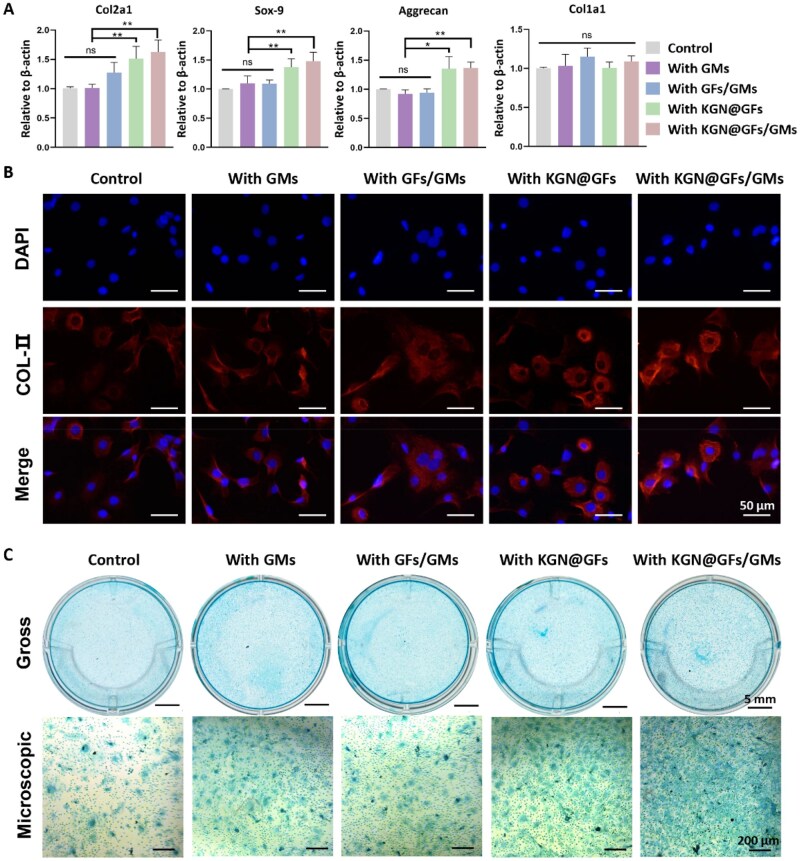
Effects of different hydrogel groups on chondrogenic differentiation of BMSCs *in vitro*. (**A**) Relative gene expression of Col2a1, Sox-9, Aggrecan and Col1a1 in BMSC after 10 days of differentiation in various hydrogels (mean ± SD; *P < 0.05, **P < 0.01, ***P < 0.001, ****P < 0.0001, ns: not significant). (**B**) Immunofluorescence (IF) observation of type II collagen (COL-II) expression in BMSCs after 10 days of co-culture with various hydrogels. (**C**) Alcian blue staining of BMSCs co-cultured with groups of hydrogels and chondrogenic differentiation after 21 days.

KGN@GFs, uniformly dispersed within the hydrogels, continuously released KGN, thereby promoting the chondrogenic differentiation of BMSCs and the formation of cartilage ECM [27]. To evaluate the *in vitro* effect of the ODex/CMCS/KGN@GFs/GMs hydrogel on BMSC chondrogenesis, we extracted mRNA from BMSCs co-cultured with different hydrogels after 10 days of chondrogenic induction. The relative expression levels of chondrogenic markers were quantified by qPCR (Figure 5A). The composite system demonstrated significantly improved chondrogenic differentiation of BMSCs compared with the ODex/CMCS hydrogel. Specifically, hydrogels with ODex/CMCS/KGN@GFs/GMs hydrogel exhibited ∼1.61-fold, 1.48-fold and 1.36-fold increases in Collagen II (Col-II), Sox-9 and Aggrecan expression, respectively, compared to the control group. In contrast, no statistically significant differences were observed in Collagen I (Col-I) expression (a marker of fibroblastic or osteogenic differentiation) across all groups. In addition to gene expression analysis, immunofluorescence (IF) staining was performed to verify Col-II expression at the protein level. As shown in Figure 5B, after 10 days of co-culture, BMSCs in the KGN-loaded groups (ODex/CMCS/GFs and ODex/CMCS/GFs/GMs) exhibited intense and widespread red fluorescence for type II collagen (Col-II). Furthermore, cells in these groups displayed typical chondrocyte-like morphologies, transitioning toward an ellipsoidal or near-spherical shape and forming distinct cell clusters. In contrast, the control groups showed only faint and localized fluorescence, indicating limited chondrogenic potential.

To further evaluate the impact of the ODex/CMCS/KGN@GFs/GMs hydrogel on cartilage matrix synthesis, Alcian blue staining was conducted using BMSCs co-cultured with different hydrogels formulations. Quantitative analysis revealed that BMSCs cultured with the ODex/CMCS/KGN@GFs/GMs hydrogel exhibited maximal glycosaminoglycans (GAGs) accumulation, corresponding to intense Alcian blue staining intensity (Figure 5C). Furthermore, GM-containing hydrogels displayed significantly increased GAG deposition compared to controls, a phenomenon primarily resulting from GM dissolution, leading to the formation of interconnected 3D porosity. This architecture augmented intercellular communication and ECM remodeling, promoted BMSC clustering and enabled efficient mass transport of nutrients/metabolites, collectively enhancing chondrogenesis and matrix biosynthesis.

In summary, these molecular and histological findings underscore the potent biochemical efficacy of the hybrid system. The significant upregulation of type II collagen, Sox-9 and aggrecan, coupled with no significant change in type I collagen expression [41], suggests that the released KGN is likely to activate the Runx1–Sox9 signaling axis [42]. This molecular guidance preferentially redirects cellular resources toward chondrogenic-specific matrix synthesis rather than fibroblastic differentiation. These observations are strongly supported by previous studies on gelatin-based inductive environments. Specifically, Sulaiman *et al*. demonstrated that cross-linked GMs promote BMSC adhesion and cartilage-related gene expression through extensive scaffold colonization [43]. Separately, Patel *et al*. found that GM-based cell delivery systems facilitate chondrocyte expansion into dense GAG-rich ECM aggregates by optimizing nutrient diffusion, significantly surpassing bulk hydrogel systems [44]. By enhancing nutrient transport and promoting cell–cell aggregation through our *in situ* pore-forming strategy, our system successfully mimics these supportive microenvironments. Combined with the “reservoir effect” of the KGN-loaded fiber network, this architecture ensures sustained biochemical induction, providing a compelling mechanistic rationale for the high-quality cartilage regeneration achieved in the subsequent *in vivo* studies.

### *In vivo* cartilage repair capability

**Figure 6:**
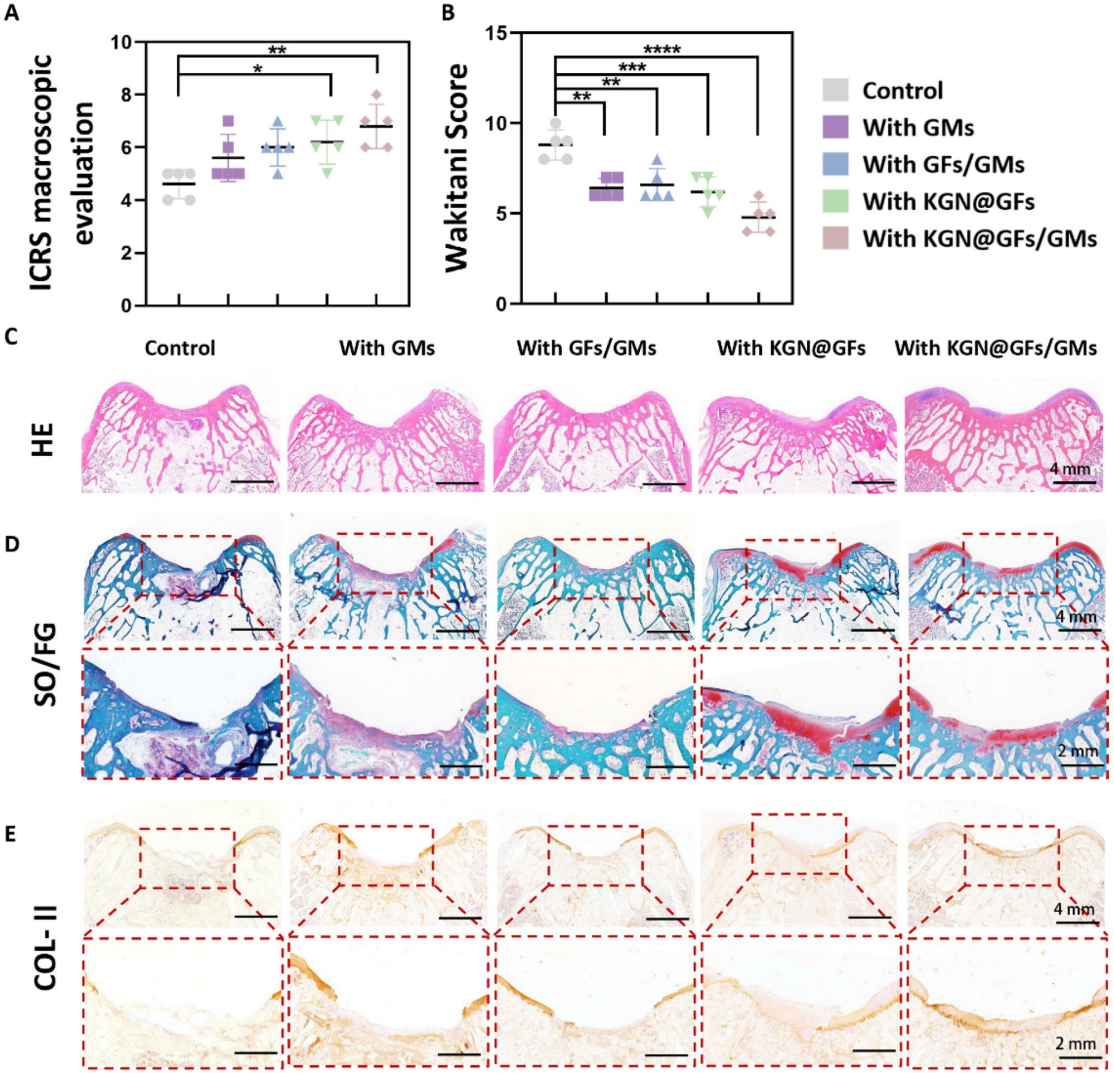
Effects of different hydrogel groups on cartilage repair in a rabbit knee defect model. (**A**) The ICRS macroscopic evaluation was used to evaluate the appearance of cartilage regeneration. (**B**) The Wakitani scoring system was used to evaluate cartilage regeneration. Histopathological evaluation of the regenerated cartilage using (**C**) hematoxylin and eosin (H&E) staining, (**D**) Safranin O–Fast Green (SO/FG) staining and (**E**) immunohistochemical staining for type II collagen.

To evaluate the translational potential of the ODex/CMCS/KGN@GFs/GMs hydrogel, we implanted five hydrogel formulations into 4-mm full-thickness femoral condyle defects in 2-month-old New Zealand White rabbits and assessed repair outcomes at 10 weeks. Macroscopic analysis using the International Cartilage Repair Society (ICRS) scoring system revealed that KGN-containing hydrogels demonstrated significantly improved defect filling, smoother surfaces and better cartilage integration with surrounding cartilage compared with ODex/CMCS hydrogel (Figure 6A and Supplementary Figure S10) [45]. In particular, the ODex/CMCS/KGN@GFs/GMs group achieved the best macroscopic appearance, reaching ICRS Grade II (nearly normal), while other experimental groups showed intermediate improvement over control.

Histological evaluation supported these macroscopic observations. H&E staining indicated more complete tissue regeneration and a smoother articular surface in KGN-containing groups (Figure 6C). Safranin O–Fast Green staining revealed substantially higher GAG content in the ODex/CMCS/KGN@GFs/GMs hydrogel group, followed by ODex/CMCS/KGN@GFs hydrogel group. Groups incorporating GMs (with or without KGN) exhibited enhanced GAG deposition versus controls (Figure 6D). Immunohistochemistry for type II collagen demonstrated stronger and more continuous Col-II immunoreactivity in the defect regions of the KGN-loaded constructs than in controls, consistent with formation of a hyaline-like matrix rather than fibrous repair tissue (Figure 6E) [46].

Furthermore, the quality of regenerated cartilage was evaluated using the scoring method described by Wakitani *et al*. based on histological criteria [47, 48]. The mean ± standard deviation (SD) scores were as follows: 8.80 ± 0.84 (control group), 6.40 ± 0.55 (ODex/CMCS/GMs group), 6.60 ± 0.89 (ODex/CMCS/GFs/GMs hydrogel group), 6.20 ± 0.84 (ODex/CMCS/KGN@GFs hydrogel group) and 4.80 ± 0.84 (ODex/CMCS/KGN@GFs/GMs hydrogel group) (Figure 6B). All four treatment groups had significantly lower scores than the control group (*P* < 0.05), with the KGN@GFs/GMs group showing the most pronounced improvement. The concordance between improved ICRS macroscopic grades and superior Wakitani histological scores indicated that the composite design enhanced repair quality at both the tissue-scale and the matrix composition level.

Mechanistically, these *in vivo* outcomes aligned with our *in vitro* findings that KGN directed BMSCs toward a chondrogenic lineage (as previously described). This process enhanced the synthesis of cartilage-specific ECM components (e.g. Col-II and aggrecan), rather than fibrocartilaginous or osteogenic matrix production (e.g. Col-I). In our model, the sustained local release of KGN from cross-linked GFs delivered a persistent pro-chondrogenic signal within the defect microenvironment, promoting hyaline-like matrix deposition and Col-II expression.

The addition of GMs produced distinct structural benefits. Groups containing GMs exhibited superior defect filling and higher GAG accumulation than their non-GMs counterparts. This effect stemmed from the porogen function of GMs: their progressive dissolution generated interconnected pores that facilitated cell infiltration and aggregation, improved nutrient and metabolite transport within the hydrogel network and provided gelatin-derived adhesive domains to promote ECM deposition. Thus, GMs address the inherent limitation of ODex/CMCS-based hydrogel: namely, limited porosity (which hinders cell ingress) and suboptimal transport capacity [49]—while KGN@GFs provided biochemical cues necessary to drive hyaline cartilage formation. The combined KGN@GFs/GMs formulation therefore yields synergistic structural and biochemical advantages that translate into superior repair.

Nevertheless, there are limitations in the current study. The rabbit trochlear groove model, while providing standardized defects for material evaluation, does not fully replicate the complex biomechanical environment of high-load-bearing regions like the femoral condyle. Additionally, the observation period was limited to 12 weeks. Future investigations will focus on long-term evaluations in large animal weight-bearing models or osteoarthritis environments to further validate the clinical translational potential of this hybrid system.

## Conclusions

Building upon the traditional ODex/CMCS hydrogel system, this study developed a structurally and functionally optimized composite hydrogel by incorporating thermosensitive GMs and KGN-loaded cross-linked GFs (KGN@GFs). The GMs functioned as pore-forming agents, generating interconnected porous structure within hydrogels to enhance nutrient diffusion, cell infiltration and tissue ingrowth. Simultaneously, the KGN@GFs provided sustained KGN release and bioactive cues through cross-linked GF network, effectively promoting chondrogenic differentiation. This synergistic design addressed the inherent limitations of conventional ODex/CMCS hydrogels—such as dense microstructure, low bioactivity and uncontrolled release—while endowing the material with enhanced structural adaptability and biological performance. *In vitro*, the pore-forming hydrogels facilitated BMSC proliferation, infiltration, aggregation and chondrogenic differentiation. *In vivo*, the ODex/CMCS/KGN@GFs/GMs hydrogel demonstrated superior cartilage repair in a rabbit defect model, yielding hyaline-like cartilage with robust integration with surrounding tissue. Collectively, these results demonstrated that combining sustained biochemical signaling with controlled structural porosity provided an effective strategy for designing advanced injectable hydrogels with improved AC regeneration potential.

## Supplementary Material

rbag060_Supplementary_Data

## Data Availability

Data will be made available on request.
