# Supplementary material for: Hybrid hydrogel system integrating thermoresponsive microspheres and KGN-loaded nanofibers enables efficient cartilage matrix regeneration
Source: Regen Biomater. 2026 Mar 21;13:rbag060. doi: 10.1093/rb/rbag060 (PMC13202449; doi:10.1093/rb/rbag060)
Supplement: rbag060_Supplementary_Data [file rbag060_supplementary_data.docx]

**Supporting Information**

**Figures:**


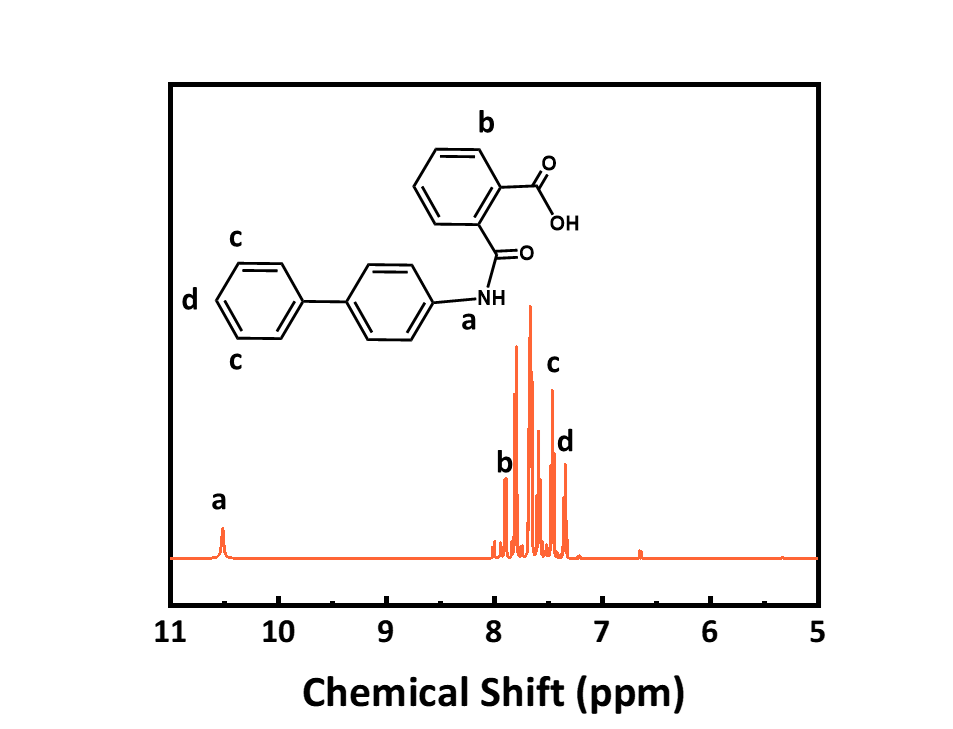


**Figure S1.** ^1^H NMR spectrum of KGN.


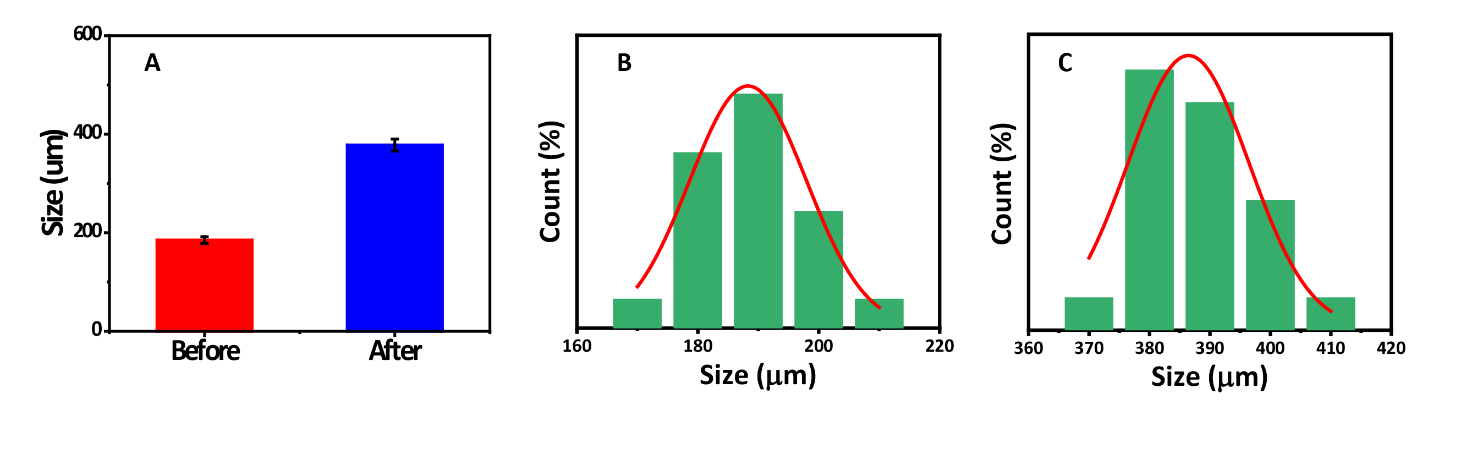


**Figure S2.** (A) Average sizes of GMs before and after swelling in PBS. Size distribution of GMs (B) before and (C) after swelling.


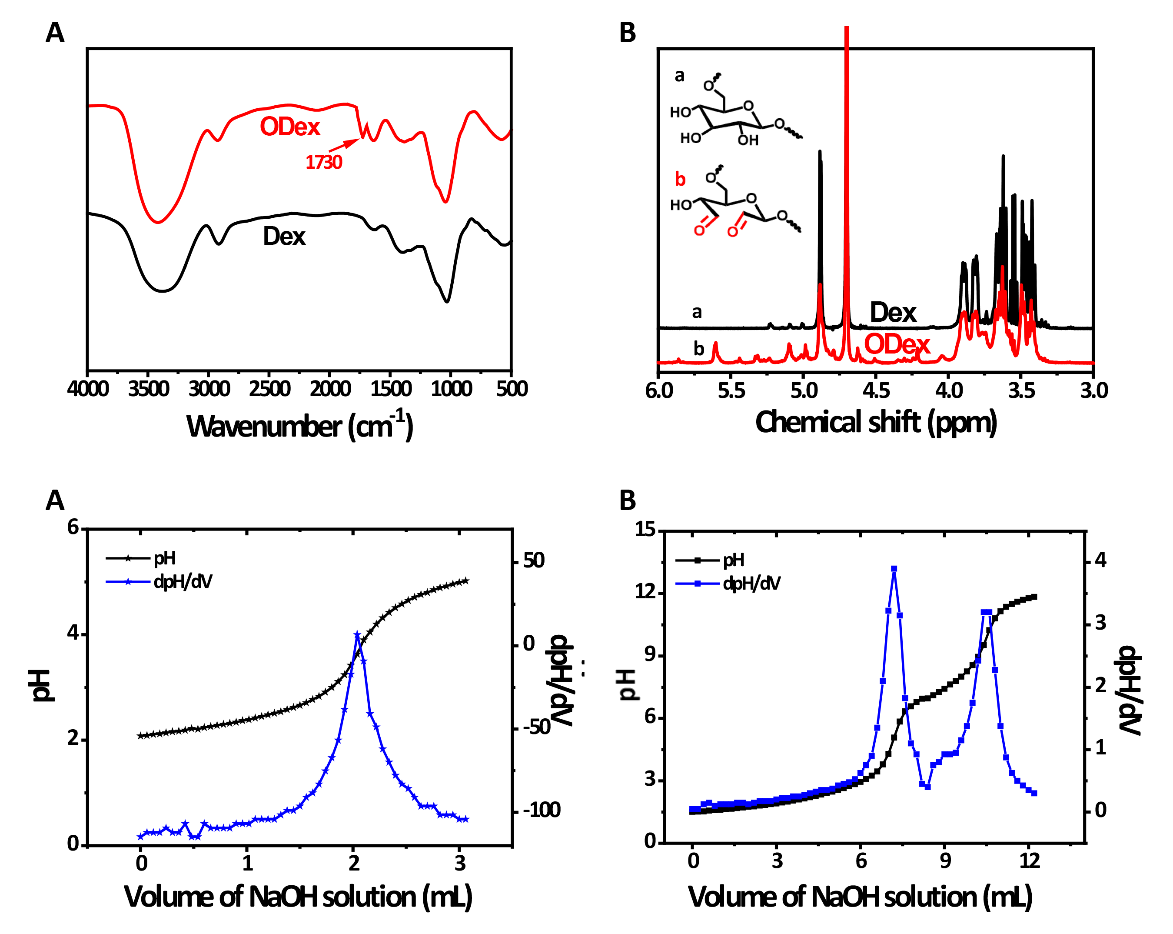


**Figure S3.** (A) FTIR and (B) ^1^H NMR spectra of Dex and ODex.


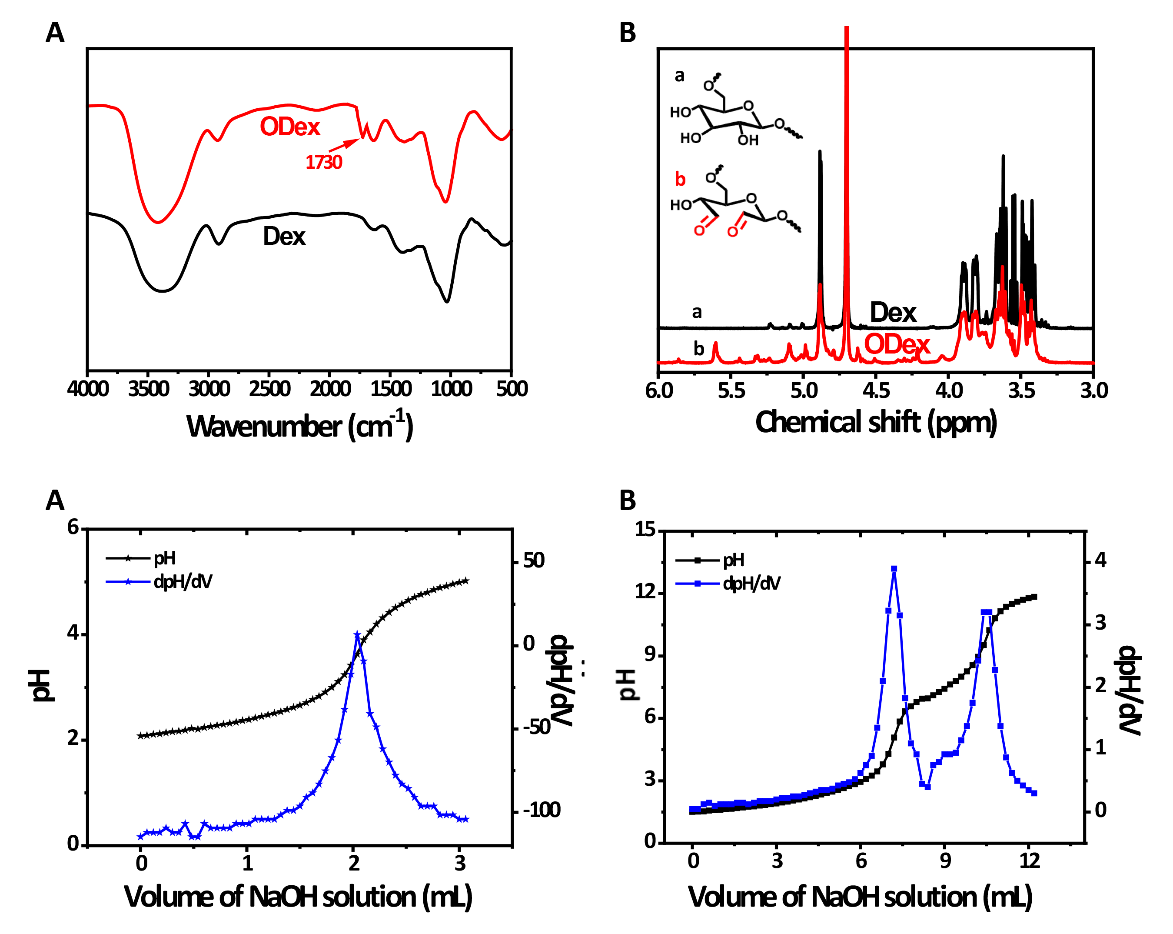


**Figure S4.** (A) Potentiometric titration of aldehyde content in ODex and its first derivative curve; (B) Potentiometric titration of amino content in CMCS and its first derivative curve.


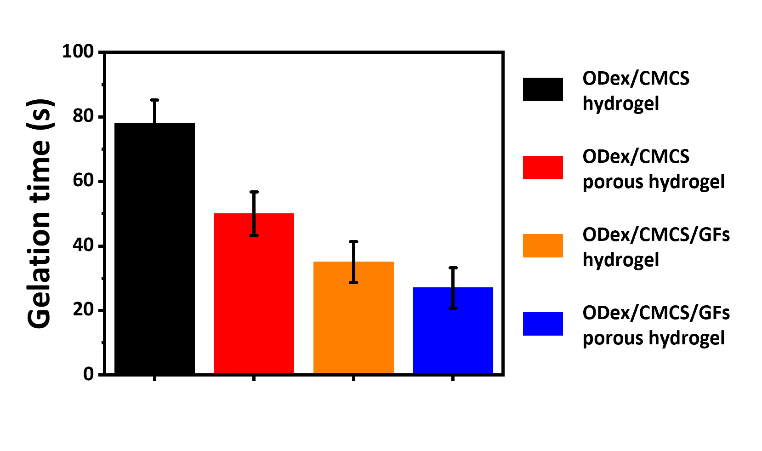


**Figure S5.** Gelation time of ODex/CMCS, ODex/CMCS/GMs, ODex/CMCS/GFs and ODex/CMCS/GFs porous hydrogels.


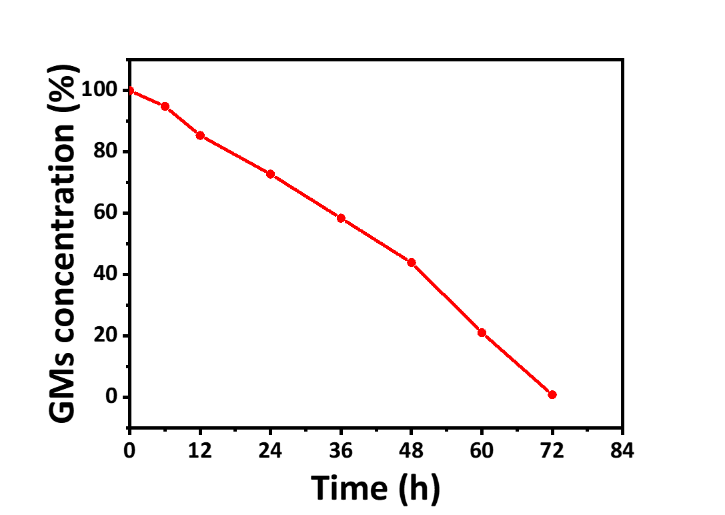


**Figure S6.** Degradation kinetics curve of the GMs.

**
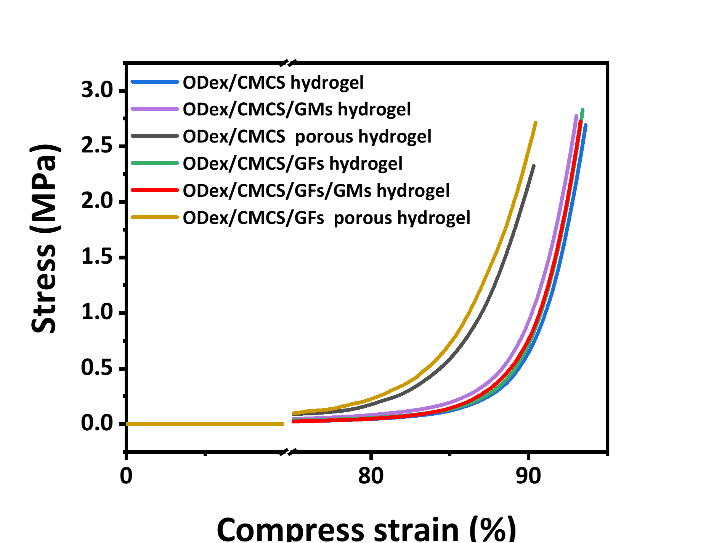
**

**Figure S7.** Compression curves of ODex/CMCS, ODex/CMCS/GMs, ODex/CMCS porous, ODex/CMCS/GFs, ODex/CMCS/GFs/GMs and ODex/CMCS/GFs porous hydrogels.


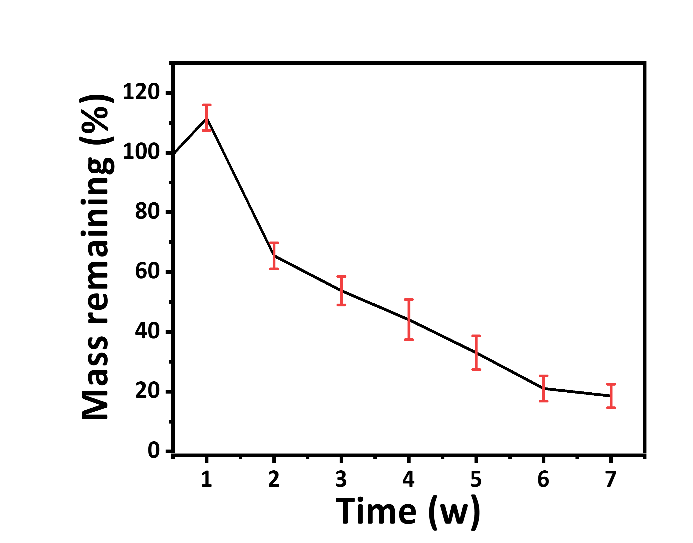
**Figure S8.** Degradation kinetics curve of the ODex/CMCS/GFs/GMs hydrogel.


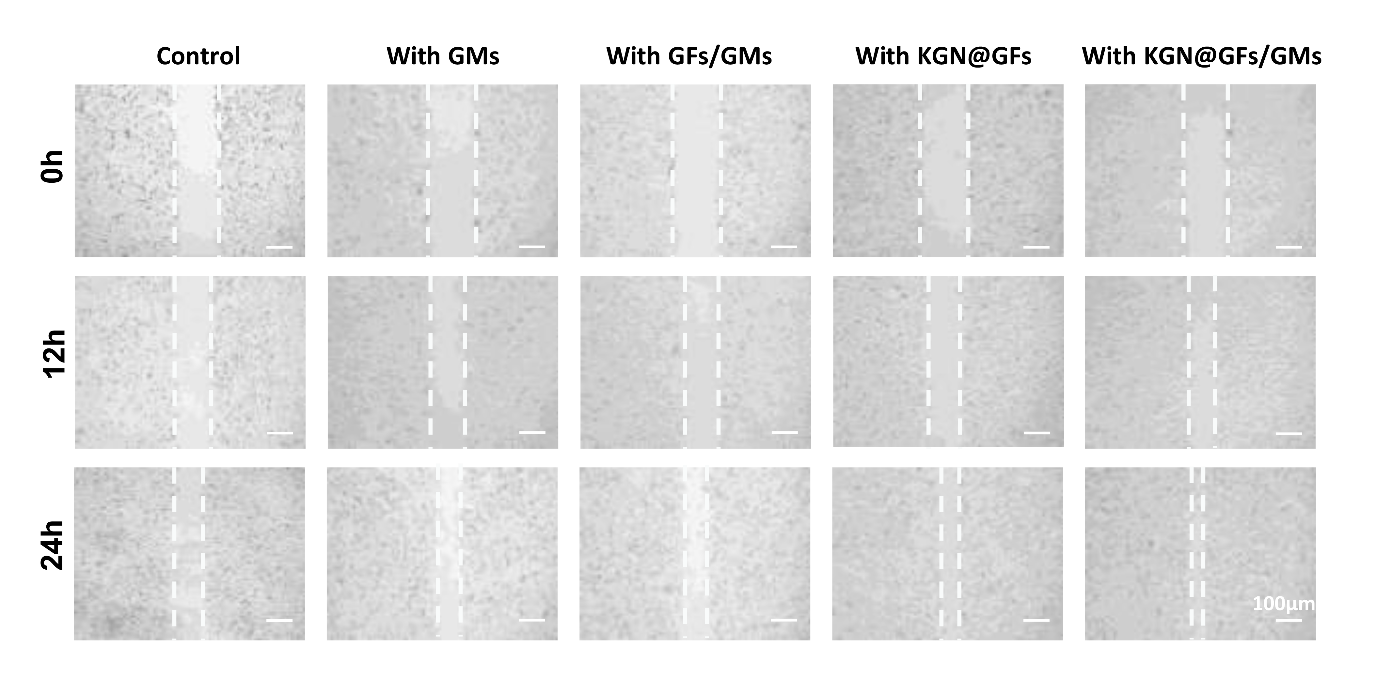


**Figure S9.** Scratch assay of BMSCs co-cultured with various hydrogels for 24 h.

**
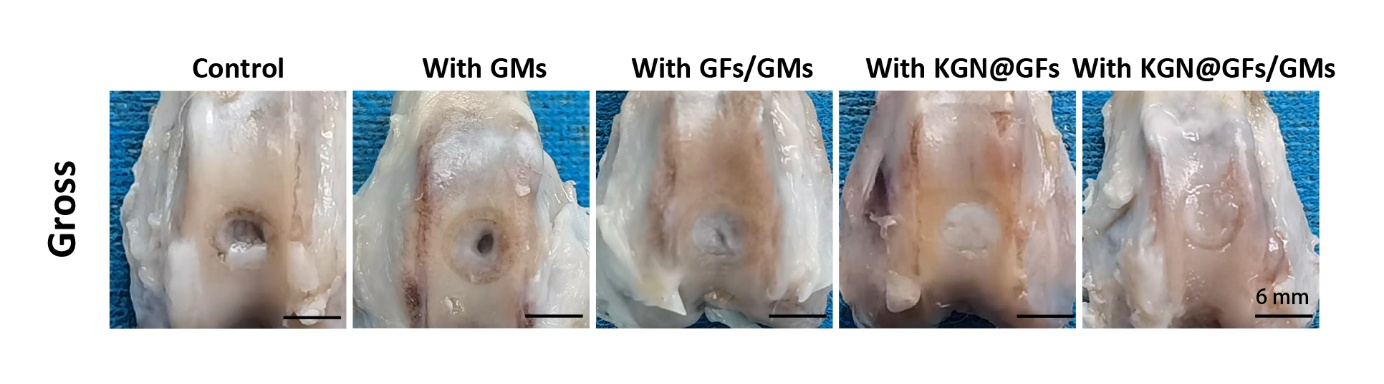
**

**Figure S10.** Gross appearance of rabbit knee cartilage defects at 10 weeks post-surgery.

**Table:**

**Table S1.** ICRS macroscopic evaluation of cartilage repair.

| **Cartilage repair assessment ICRS** | **Score** |
| --- | --- |
| **A) Degree of defect repair** |  |
| In level with surrounding cartilage | 4 |
| 75% repair of defect depth | 3 |
| 50% repair of defect depth | 2 |
| 25% repair of defect depth | 1 |
| 0% repair of defect depth | 0 |
| **B) Integration to border zone** |  |
| Complete integration with surrounding cartilage | 4 |
| Demarcating border < 1 mm | 3 |
| 3/4th of graft integrated, 1/4th with a notable border > 1 mm width | 2 |
| 1/2 of graft integrated with surrounding cartilage, 1/2 with a notable border > 1 mm | 1 |
| From no contact to 1/4th of graft integrated with surrounding cartilage | 0 |
| **C) Macroscopic appearance** |  |
| Intact smooth surface | 4 |
| Fibrillated surface | 3 |
| Small, scattered fissures or cracks | 2 |
| Several, small or few but large fissures | 1 |
| Total degeneration of a grafted area | 0 |
| **Overall repair assessment** |  |
| Grade I: normal | 12 |
| Grade II: nearly normal | 11-8 |
| Grade III: abnormal | 7-4 |
| Grade IV: severely abnormal | 3-1 |

**Table S2.** Modified Wakitani Histological Scoring System for Accessing Cartilage Repair**.**

| **Category points** | **Scores** |
| --- | --- |
| **A) Cell morphology** |  |
| Hyaline cartilage | 0 |
| Mostly hyaline cartilage | 1 |
| Mostly fibrocartilage | 2 |
| Mostly noncartilage | 3 |
| Noncartilage | 4 |
| **B) Matrix staining with safranin - O and fast green** |  |
| Normal (compared with host adjacent cartilage) | 0 |
| Slightly reduced | 1 |
| Markedly reduced | 2 |
| No metachromatic stain | 3 |
| **C) Thickness of cartilage** |  |
| > 2/3 | 0 |
| 1/3 - 2/3 | 1 |
| < 1/3 | 2 |
| **D) Integration of implant with adjacent host cartilage** |  |
| Both edges integrated | 0 |
| One edge integrated | 1 |
| Neither edge integrated | 2 |
| **Total maximum** | 11 |

**Table S3.** Compositions of various hydrogels.

| Hydrogels | ODex/CMCS | GMs | GFs | KGN@GFs | Name |
| --- | --- | --- | --- | --- | --- |
| Control (ODex/CMCS) | √ |  |  |  | Control |
| ODex/CMCS /GMs | √ | √ |  |  | With GMs |
| ODex/CMCS/GFs/GMs | √ | √ | √ |  | With GFs/GMs |
| ODex/CMCS/KGN@GFs | √ |  |  | √ | With KGN@GFs |
| ODex/CMCS/KGN@GFs/GMs | √ | √ |  | √ | With KGN@GFs/GMs |

Note: In biological studies (at 37 °C), the GMs-containing groups represent the *in situ* pore-formed state, as the GMs dissolve automatically under physiological conditions.
